# Wastewater Surveillance to Track Resurgent Measles Outbreak, Ontario, Canada, 2025

**DOI:** 10.3201/eid3210.260092

**Published:** 2026-10

**Authors:** Ryland Corchis-Scott, Élisabeth Mercier, Edgard M. Mejia, Qiudi Geng, Ethan Harrop, Ana Podadera, Jackie Fong, Natalie Lewoc, Kenneth K.S. Ng, Nataliya Santiago, Natalie Knox, Lawrence Goodridge, Chand S. Mangat, Chrystal Landgraff, Karen B. Riddell, Mehdi Aloosh, Robert Delatolla, R. Michael McKay

**Affiliations:** University of Windsor, Windsor, Ontario, Canada (R. Corchis-Scott, Q. Geng, E. Harrop, A. Podadera, J. Fong, K.K.S. Ng, R.M. McKay); University of Ottawa, Ottawa, Ontario, Canada (É. Mercier, R. Delatolla); National Microbiology Laboratory, Winnipeg, Manitoba, Canada (E.M. Mejia, N. Santiago, N. Knox, C.S. Mangat, C. Landgraff); Windsor Essex County Health Unit, Windsor (N. Lewoc, M. Aloosh); University of Guelph, Guelph, Ontario, Canada (L. Goodridge); Windsor Regional Hospital, Windsor (K.B. Riddell); McMaster University, Hamilton, Ontario, Canada (M. Aloosh)

**Keywords:** wastewater, measles, viruses, public health, vaccines, wastewater surveillance, vaccination hesitancy, vaccine-preventable diseases, sewage, incidence, epidemiology, Canada

## Abstract

The province of Ontario, Canada, experienced a major resurgent outbreak of measles in 2025. We conducted wastewater surveillance concurrently with clinical-based surveillance to track measles incidence in southwestern Ontario, adjacent to the United States. Measles virus (MeV) signal in wastewater was positively associated with clinical cases but did not provide early alert of changes in measles incidence when resolved by epidemiologic week. Assessment of virus partitioning showed MeV RNA was broadly distributed in the liquid phase but is most concentrated in the solids. We adapted an assay for differentiation of vaccine and wild-type MeV and used it to detect vaccine genotype measles after a vaccination campaign targeting underserved groups. We estimated MeV shedding in wastewater through sampling of sewer laterals serving a hospital treating measles infections. This outbreak is a case study in which we highlighted the use of wastewater surveillance for measles while supporting method development in real time.

Wastewater surveillance (WS) gained traction during the COVID-19 pandemic as an effective and unbiased means to track community infection complementary to clinical testing. Sampling a wastewater treatment plant (WWTP) or locations within a sewershed revealed infection hotspots and tracked genetic variants (*1*). Wastewater yielded high-quality whole-genome SARS-CoV-2 sequence data (*2*); it is a resource for tracking disease spread and a complement to clinical data to identify disease burden and routes of pathogen transmission (*3*,*4*). WS has extended to additional targets, including respiratory diseases (*5*), foodborne illness (*6*), and diseases of emerging concern, such as mpox and highly pathogenic avian influenza (*7*–*9*). Public health officials warned of the increased potential for outbreaks of vaccine-preventable diseases (*10*). COVID-19 pandemic–associated disruptions and vaccine misinformation affected immunization campaigns, including measles vaccination programs (*11*). 

In 2025, Ontario experienced a large outbreak of measles; ≈2,400 confirmed cases were reported. Total cases nationwide reached 5,461 in 2025, positioning Canada as a hotspot for measles in North America (*12*). In November 2025, after 12 months of sustained transmission, Canada lost measles elimination status (*13*), a designation attained in 1998 (*14*). The 2025 outbreak of measles in Canada likely resulted from reduced vaccination coverage (*15*) and was the largest outbreak in 3 decades, overwhelming public health services, especially in southwestern Ontario, which experienced the highest caseload (*16*). Concurrent outbreaks in the US Southwest (*17*) and in Alberta, Canada (*18*), have raised interest in WS to complement clinical testing (*19*). 

WS accounts for community members who might be hesitant to seek medical care (*20*); that characteristic is relevant to measles, considering most cases in North America have been linked to close-knit undervaccinated communities (*21*,*22*). Studies since 2025 have highlighted the application of WS to track measles at a community level (*23*–*26*) and assays that differentiate between wild-type virus and vaccine genotypes (*20*,*27*). Despite early successes, many challenges persist to broader and more actionable adoption, including the clinical dynamics of viral shedding, viral partitioning within wastewater, and detection sensitivity (*21*), as well as the proximity of cases to monitored sewersheds. Here, we present a case study from a rural border municipality in southwestern Ontario demonstrating the application of WS for MeV during a measles outbreak. The outbreak persisted for >9 months, affording an opportunity for WS method development and enabling investigation to produce insights on MeV partitioning and on rates of viral shedding in wastewater. Our investigation used the outbreak as an opportunity to determine the feasibility of WS for MeV in a semirural setting and to help address challenges to its implementation.

## Methods

### Sample Collection

As part of an ongoing WS program, we collected composite wastewater samples over 24-hour periods, 3 per week, from the Leamington Pollution Control Centre (LPCC) in the Windsor-Essex region of Ontario, Canada, during February–November 2025. The LPCC serves the urban center of Leamington and accepts septage from residential septic tanks and frequently emptied storage tanks serving congregate living facilities in the rural area (≈30,000 total population served). We monitored sewer laterals draining Windsor Regional Hospital (WRH) using passive samplers (tampons) deployed 1–2 times/week. Passive samplers were submerged in the hospital effluent stream for 17–24 hours; at collection, they were transported to the laboratory for immediate processing (*28*).

### Sample Processing for MeV Monitoring

We concentrated wastewater influent samples by filtration or affinity capture methods, and hospital effluent samples by centrifugation. After concentration, we extracted total nucleic acids and measured viral concentrations with quantitative reverse transcription PCR (qRT-PCR). We used an assay targeting the MeV nucleoprotein (N) gene for measles quantification (Appendix Table 1). We used a separate assay to measure pepper mild mottle virus in samples (*28*). We conducted sequencing to validate the identity of the amplicons obtained from the N gene of MeV (Appendix).

### Partitioning of Endogenous MeV in Wastewater

To determine the partitioning of endogenous MeV in wastewater, we processed a 4-L postgrit wastewater sample collected from LPCC to derive fractions of solids, colloids, and supernatant (Figure 1). We performed partitioning calculations using 3 different assumptions: whole-sample basis, which considered wastewater composition; equal-mass basis; and operational volume basis, which reflects processing volumes commonly used in laboratory workflows (Appendix).

**Figure 1:**
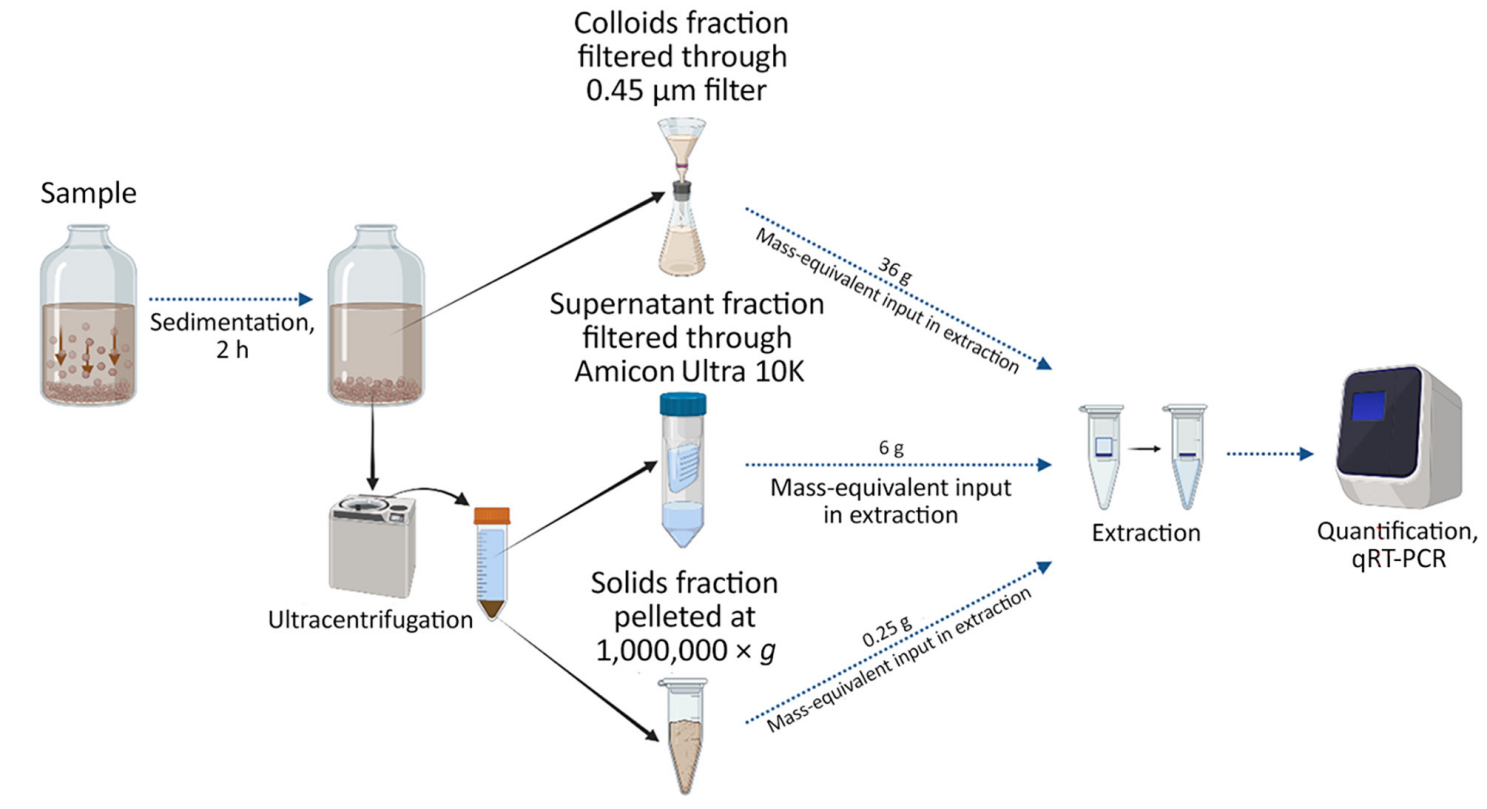
Schematic overview of wastewater processing in study of the utility of wastewater surveillance to track a resurgent outbreak of measles, Ontario, Canada, 2025. Sample processing and fractionation protocol were used to partition endogenous measles virus into solids, colloids, and supernatant fractions using Amicon Ultra 10K filters (Sigma Aldrich, https://www.sigmaaldrich.com) in wastewater collected from Leamington, Ontario, Canada.

### Detection of Vaccine Genotype MeV

We shipped raw influent samples weekly to the National Microbiology Laboratory (Winnipeg, MB, Canada) for additional analysis. Samples were processed as described previously (*29*), with minor modifications. To measure MeV wild-type and vaccine genotypes in wastewater, we used a multiplex assay developed for clinical detection (*30*). That triplex assay targets the large (L) gene for pan-MeV detection and the hemagglutinin (H) gene for wild-type and vaccine differentiation using a locked nucleic acid (LNA) probe for each target (Appendix).

### Additional Data Sources

The WRH provided deidentified hospital admission and discharge dates for patients admitted with measles along with hospital water usage data. We obtained weekly measles case data from the Windsor-Essex County Health Unit (WECHU). We deemed symptomatic persons who sought care for fever; maculopapular rash for >3 days; and cough, coryza, or conjunctivitis, along with laboratory confirmation of infection, as having confirmed cases of measles. We also considered clinically compatible signs and symptoms in a person with an epidemiologic link to a laboratory-confirmed measles case (*31*). The WECHU provided weekly vaccination data consisting of the number of measles, mumps, and rubella (MMR) vaccines administered in a targeted vaccination campaign. LPCC provided metadata (pH, temperature, flow) for each sample and the septage volume deposited at LPCC by private companies. The Windsor Regional Hospital Research Ethics Board (REB no. 25-529) and the University of Windsor Research Ethics Board (REB no. 25-127) provided research ethics approval.

### Estimation of Viral Shedding

We estimated viral shedding rates for MeV using passive samplers deployed at WRH as described previously (*28*), with slight modifications. Because it is challenging to measure viral gene concentration in sampled flows using passive samplers, we calculated the concentration of MeV in hospital effluent using a previously reported mean suspended solids value for hospital effluent (*32*) and accounting for MeV partitioning. We determined flow rates using hospital water use. We used admission and discharge times for MeV hospitalizations to determine both the number of hospitalized patients and the temporal overlap value. We reported shedding rates in log10 gene copies per day per person. We used median estimated shedding rate, average weekly flow, and MeV concentration to create an estimated weekly case count for LPCC sewershed (Appendix).

### Statistical Methods

We performed statistical analyses using R version 4.5.1 (The R Project for Statistical Computing, https://www.r-project.org). We analyzed data collected during February–July 2025, ending with the last reported measles case in Leamington (Appendix).

## Results 

### Partitioning of MeV in Wastewater

We determined the distribution of endogenous MeV across settled solids, colloids, and supernatant fractions of wastewater. We calculated MeV distribution using 3 different approaches (Figure 2). When accounting for typical wastewater composition (99.96% liquid and 0.04% solids), we detected most of the MeV signal (+SD) in the supernatant (91.20% +7.22%), followed by the colloids (8.58% +1.24%) and settled solids (0.22% +0.06%) (Figure 2, panel A). That approach was influenced by the disproportionate volume of the liquid phase and might not reflect the concentration potential of each matrix. On an equivalent-mass basis (1 g each of solids and liquids), we found the highest concentration of MeV in the solids (85.43% +15.24%), with lower proportions in supernatant (13.31% +1.05%) and colloids (1.25% +0.18%) (Figure 2, panel B). However, routine enrichment methods do not process equivalent masses, which limited the relevance of our comparison. We used weight and volume units typically processed in laboratory workflows (0.25 g for solids, 150 mL for colloids, and 37 mL for supernatant) for the final calculation. Under operational conditions, we recovered most of the MeV signal from the supernatant (70.18% +5.55%), followed by colloids (26.77 +3.87%), and solids (3.04% +0.79%) (Figure 2, panel C). Those findings demonstrated that MeV RNA was broadly distributed in the liquid phase but was most concentrated in the solids. However, when considering viral recovery in accordance with standard enrichment protocol, the supernatant yields the highest amount of MeV RNA because it contains the largest volume, supporting its use for optimal detection.

**Figure 2:**
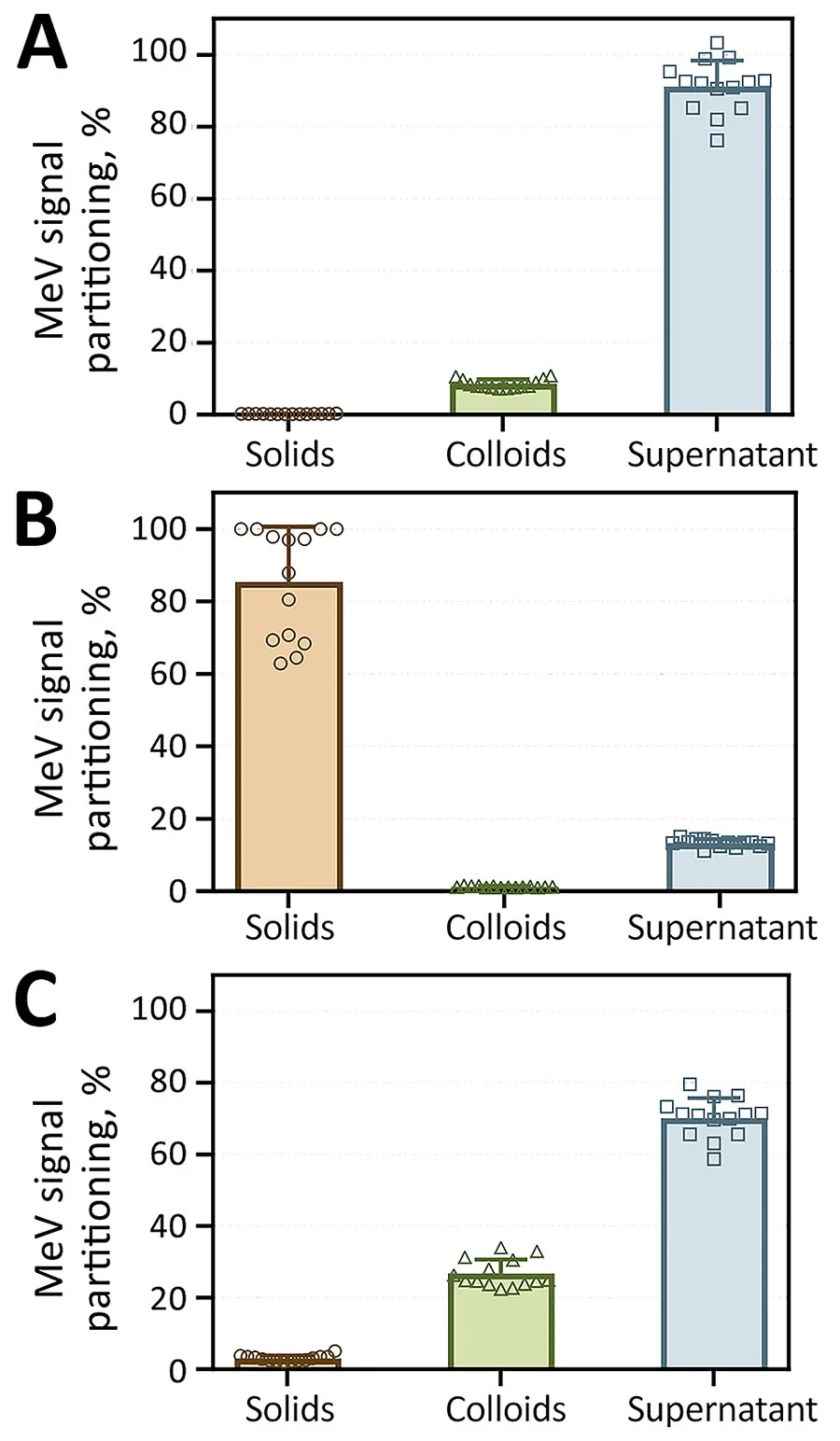
Partitioning of MeV signal across wastewater fractions in a study of wastewater surveillance conducted in Leamington, Ontario, Canada, during the 2025 outbreak. Partitioning of measles virus is shown for 3 calculation bases. Each point represents 1 technical triplicate for each of the biologic replicates (n = 14). For each bar, the upper border of each bar indicates the mean; whiskers indicate standard error. Where the standard deviation is too small, the error bars are not displayed. A) Signal partitioned assuming the composition of a typical wastewater sample (99.96% liquid, 0.04% solids). B) Signal partitioned on an equivalent mass basis, assuming 1 g of each fraction. C) Signal partitioned based on typical laboratory processing volumes: 0.25 g for settled solids, 150 mL for the colloids fraction, and 37 mL for the supernatant. MeV, measles virus.

### Wastewater Surveillance of MeV at Outbreak Location

WS of MeV began in March 2025, after reports of cases in Windsor-Essex. We conducted retrospective analysis of archived samples from February onward. The first wastewater samples that tested positive for MeV were collected on March 7, 2025 (Figure 3); samples tested positive through mid-August 2025. MeV wastewater signal showed a strong positive correlation with cases during February–July 2025 for normalized (Spearman correlation coefficient [ρ] = 0.77 [95% CI 0.51–0.87]; p<0.001) and raw (ρ = 0.77 [95% CI 0.49–0.90]; p<0.001) signal. Correlations between wastewater signal derived from a subset of samples concentrated with affinity capture techniques corroborated that result for normalized (ρ = 0.84 [95% CI 0.60–0.94]; p<0.001) and raw (ρ = 0.87 [95% CI 0.68–0.95]; p<0.001) signal. The increased correlation for that sample set could result from the truncated dataset or the use of affinity capture methods, which are more effective than filtration in concentrating MeV. We observed the correlation even where populations might underutilize health services, which likely contributed to underreporting of measles cases (*33*). We confirmed the identity of amplicons obtained by qRT-PCR by sequencing. Sequence alignments determined using BLAST (https://blast.ncbi.nlm.nih.gov/Blast.cgi) confirmed that the reads we obtained shared ≥99% identity with MeV N gene.

**Figure 3:**
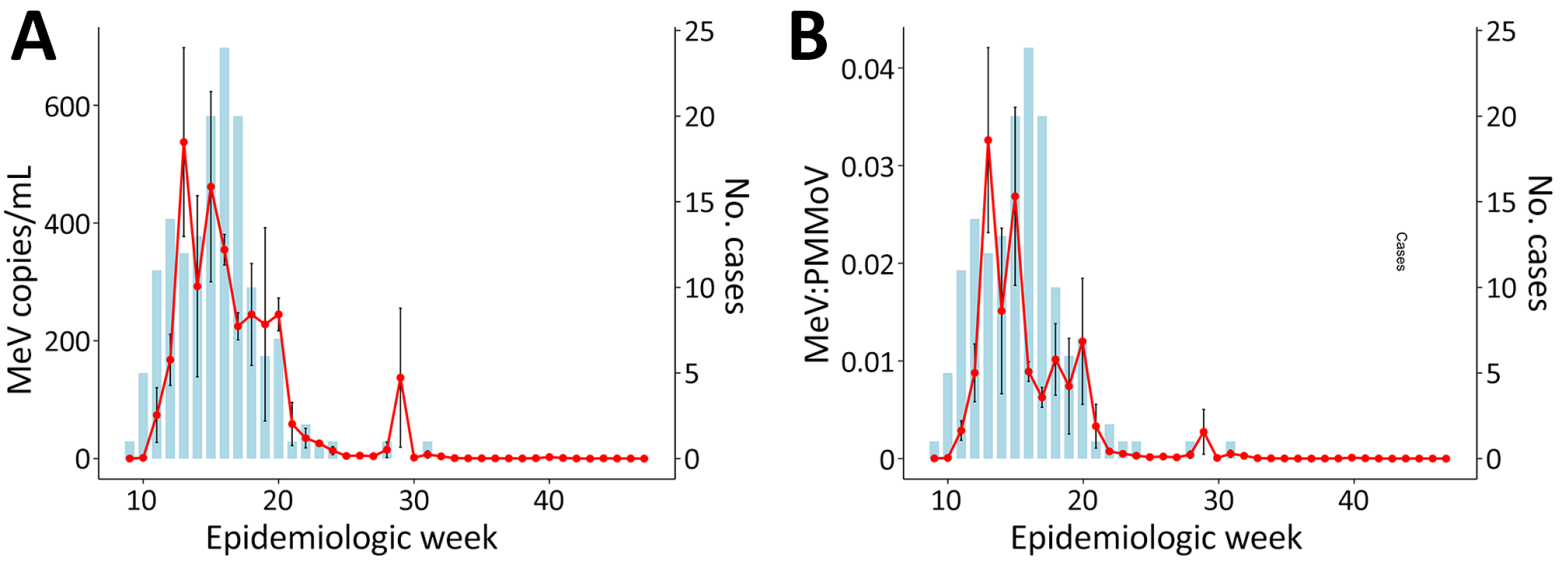
MeV wastewater surveillance signal and clinical case counts in Leamington, Ontario, outbreak, 2025. Clinical cases (blue bars) are compared against (A) raw MeV RNA concentrations (copies/mL; red points/line) and (B) MeV RNA concentrations normalized to PMMoV (red points/line). Data are aggregated by epidemiologic week; wastewater values represent weekly mean (points) + SE. MeV, measles virus; PMMoV, pepper mild mottle virus.

Previous research has shown WS to be a leading indicator of disease incidence (*34*–*38*). We used time-lagged cross-correlation (TLCC) to determine if WS for measles is a leading indicator of clinical reporting. We found the highest association when MeV wastewater signal lagged clinical cases by 1 week for pepper mild mottle virus normalized and raw signal (Appendix Figures 1, 2). However, correlations at a lag of 1 week were not different from the correlations of aligned time series (ρ = 0.79 for raw and ρ = 0.78 for normalized signal). TLCC suggested that wastewater was not a leading indicator of measles cases for data aggregated by epidemiologic week. Weekly averaging or potential delays in the detection of MeV signals at LPCC associated with septic hauling could have obscured lead time. The WS approach failed to warn of outbreak onset, potentially because of RNA degradation in archived samples (*39*), 3 times weekly sampling frequency, method sensitivity, or the rural nature of the sewershed where the outbreak occurred. 

### Modeling Determinants of MeV RNA Concentration in Wastewater Samples

The gamma generalized linear model (GLM) explained 75% of the variation in normalized MeV (pseudo R^2^ = 0.75). Clinical cases were the only statistically significant predictor. Cases had a positive influence on MeV signal (β = 2.05 +0.77; z = 2.67; p = 0.008); an increase in cases by 1 SD resulted in ≈8-fold increase in normalized MeV. Other fixed effects (flow, pH, epidemiologic week, and vaccination rates) were not statistically significant (Appendix Table 2).

We fitted a separate gamma GLM to a truncated dataset (n = 14), including cases and total sewage volume as predictors of normalized measles concentration (Appendix Table 3). The model explained 72% of variability in normalized MeV concentration (pseudo R^2^ = 0.72). Residuals simulated in DHARMa (https://CRAN.R-project.org/package=DHARMa) showed evidence of mild heteroskedasticity (Appendix Table 3). Analysis of the truncated dataset corroborated that cases are the most significant factor explaining variability in normalized MeV concentration at the WWTP; that finding reinforced the use of WS as an independent measure of disease within a community. The total volume of sewage deposited at the WWTP was a significant predictor of MeV signal. Septic sewage could contain MeV RNA, contributing to MeV signal at the WWTP when tanks are emptied frequently. Delivery delays the arrival of viral material to the WWTP and likely contributes to the observed lag at epidemiologic week temporal granularity (Appendix Figure 3). We ran Spearman correlations between physicochemical parameters of wastewater, MeV signal, and the total volume deposited by septic sewage hauling companies each week; those analyses showed that the volumes deposited by select companies and MeV signal were correlated (Appendix Figure 4). Total volume for company 3 was correlated with both normalized (ρ = 0.83; p<0.001) and raw (ρ = 0.78; p = 0.002) MeV signal in wastewater. We also observed a correlation between the total volume of all septage deposited and both raw (ρ = 0.78; p = 0.002) and normalized (ρ = 0.81; p<0.001) MeV signal. Those correlations indicated that septic hauling might contribute to the MeV signal at LPCC. We tested the association on a dataset that included only days when septage deliveries coincided with wastewater sample collection (Appendix Figure 5). Correlations were weaker and nonsignificant when restricted to days with coincident sampling, (Appendix Figure 6). Thus, septic deliveries play a more minor role in explaining wastewater signal than the first correlation analysis suggested.

### Vaccine Genotype Detection

We performed an assay that targets a single-nucleotide polymorphism (SNP) in the MeV H gene to distinguish vaccine genotype MeV RNA from wild-type MeV RNA (*30*). We treated the assay as a binary indicator of the presence of vaccine genotype MeV RNA. We detected vaccine genotype MeV in Leamington wastewater ≈6 weeks after the start of vaccination campaigns and ≈4 weeks after the intensification of the vaccination campaign (Figure 4). Research in MeV shedding dynamics after vaccination indicates that shedding can start >1 day after inoculation and can persist for up to 14 days in urine and 29 days in nasopharyngeal swabs (*40*,*41*). Those values partially explain the delay between the start of the vaccination campaign and the first detections in wastewater but are unlikely to account for the full lag. In this outbreak, the vaccination campaign focused on agri-food workers in bunkhouses served by frequently emptied septic tanks; thus, the delayed delivery of MeV RNA to the WWTP could explain the delay. The frequency of septic hauling could contribute to the temporal dynamics of WS for rural sewersheds.

**Figure 4:**
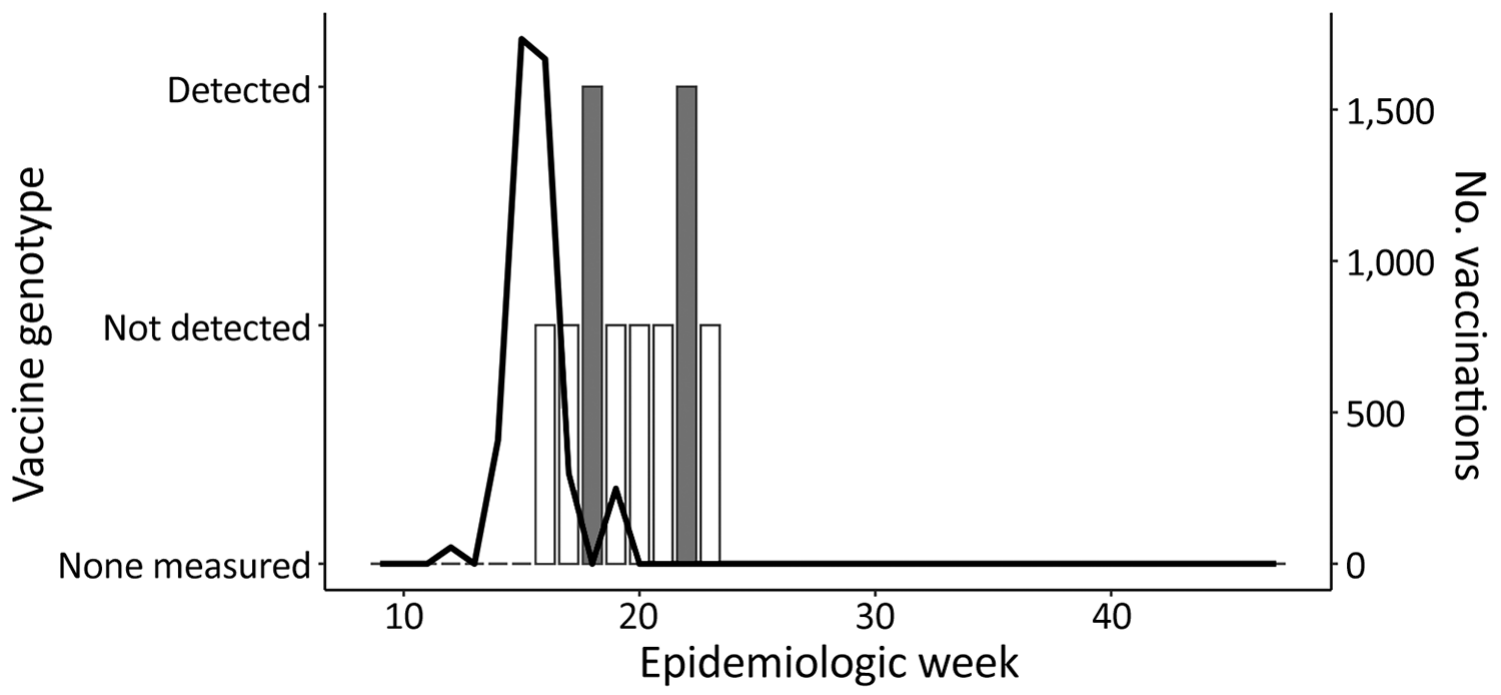
Wastewater surveillance for vaccine genotype measles and vaccination data for measles virus in Leamington, Ontario, 2025. Black line indicates weekly vaccine administrations and trend; dark bars indicate wastewater detections of MeV hemagglutinin gene single-nucleotide polymorphism unique to the vaccine genotype. White bars indicate weeks in which vaccine genotype measles virus was not detected.

### MeV Shedding and Case Estimation

The city of Windsor was less affected by the measles outbreak, consistent with higher vaccination rates in urban centers (*42*,*43*). Data from cases and wastewater signal were sporadic; each supported lower incidence in Windsor (data not shown). Simultaneous detections in hospital effluent at a campus of WRH and hospitalizations enabled us to estimate viral shedding rates on 6 dates (Table). Shedding rates ranged over 4 orders of magnitude; median shedding rate was 1.41 × 10^11^ gene copies/person/day. For 1 hospitalized patient, we estimated shedding on consecutive days. Shedding nominally decreased between measurements, but that decrease could be measurement error. Studies have shown that shedding rates of measles vary over the course of infection and between patients, generally declining in titers over time (*44*,*45*). The declining trend was not consistently supported; another study found no difference in MeV RNA concentration between early or intermediate oral fluid or serum samples (*46*). Shedding rates varied between patients and excreta type (*47*); urine displayed relatively high MeV concentrations (*21*). MeV RNA levels did not differ by vaccination status or age (*46*,*48*). 

**Table:** Daily measles virus shedding estimates based on samples collected from hospital effluent in Windsor, Ontario, Canada, March–May 2025*

Date	No. patients hospitalized	Shedding estimate, gc/d/person, +SD
Mar 25	2	1.53 × 10^12^ +2.64 × 10^11^
Mar 26	2	6.60 × 10^12^ +4.13 × 10^11^
Apr 8	1	3.73 × 10^8^ +1.47 × 10^8^
Apr 9	1	3.32 × 10^8^ +1.59 × 10^8^
Apr 22	2	7.61 × 10^8^ +7.02 × 10^8^
May 6	1	4.17 × 10^11^ +9.73 × 10^10^

*gc, gene copies.

Our estimates of MeV shedding rates were higher than previous estimates (*21*). However, given multiple sources of excreta contribute to the wastewater signal and high variability in MeV RNA measurements previously reported (*44*), the shedding estimates we derived seem reasonable. In addition, when we used the calculated median of shedding rate to extrapolate the number of measles cases in Leamington on the basis of the wastewater signal, we observed general agreement (Figure 5). Public health officials estimated the true incidence of measles in the Leamington outbreak to be 5–10 times higher than reported cases (M. Aloosh, pers. comm., email, 2025 Oct 3). That estimate is consistent with MeV outbreaks in undervaccinated communities in the Netherlands (*49*).

**Figure 5:**
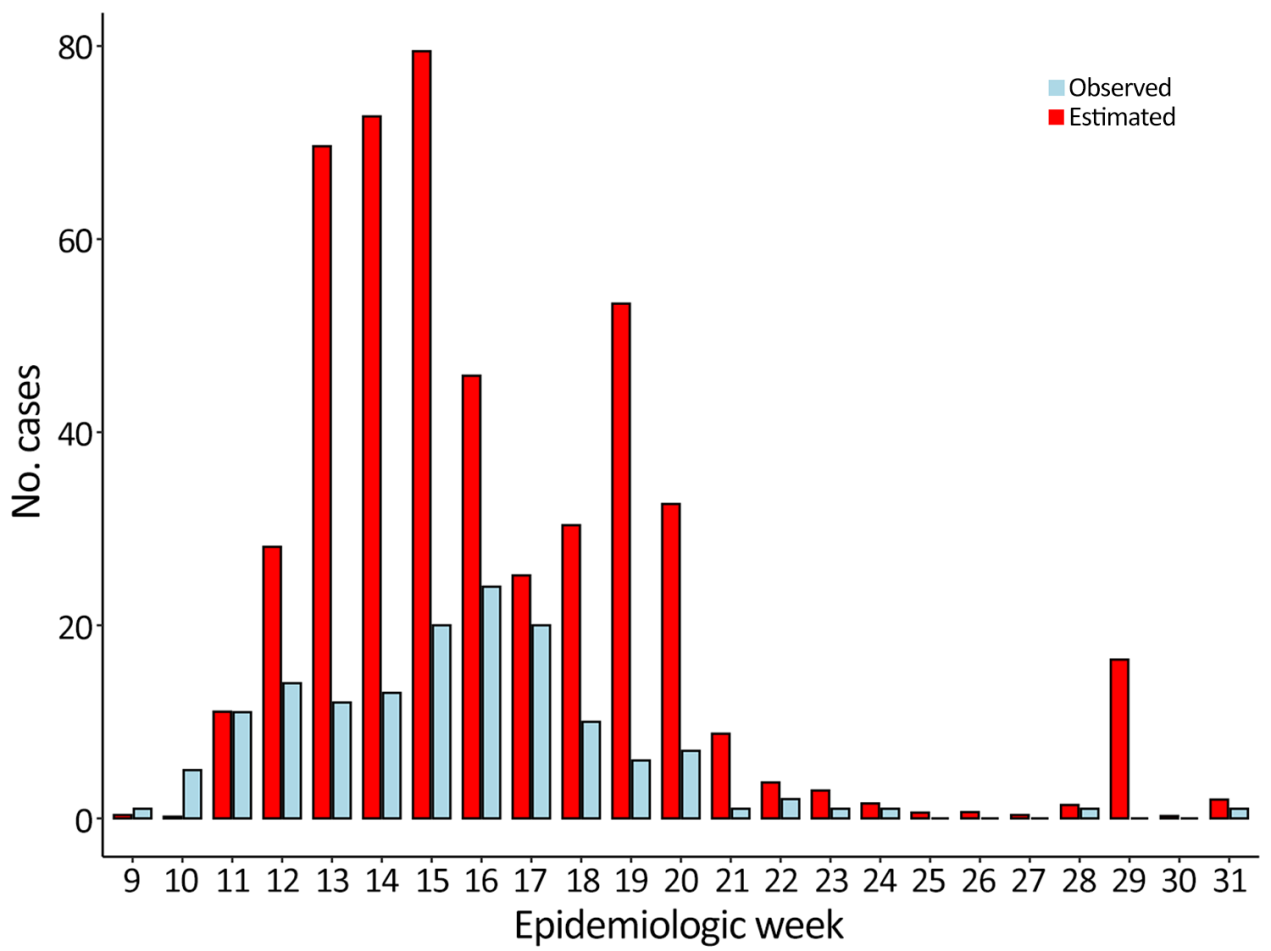
Measles case estimates for the municipality of Leamington, Ontario, Canada, based on wastewater signal and measles virus shedding approximations generated in Windsor, Ontario. Case estimations are based on median shedding rate approximations. Blue bars represent observed cases; red bars represent estimated cases.

## Discussion 

WS has the potential to become an important complementary public health tool, particularly for identifying MeV transmission in populations that are undervaccinated or less likely to access healthcare. Case-based surveillance alone might underestimate transmission (*49*). WS also provides more timely situation awareness during outbreaks, particularly when delays in reporting or low healthcare utilization might hamper clinical surveillance. WS could provide advanced warnings for changes in disease incidence during a respiratory season, outbreak, or epidemic, and can also warn of the onset of an outbreak or respiratory season (*6*,*38*). WS could be useful for early detection of MeV outbreaks because infected persons shed RNA during the prodromal period, before rash onset, whereas clinical surveillance generally identifies cases after symptom onset and laboratory confirmation (*24*). Studies have also shown that WS can provide early warning for MeV outbreaks (*25*,*26*). Early outbreak detection enables rapid mobilization of public health resources, including expedited testing, contact tracing, isolation of suspected cases, and targeted vaccinations; such actions can reduce transmission and infections, which is especially important for highly contagious pathogens such as MeV. 

In our investigation, WS did not provide warning of MeV circulation or outbreak onset before routine case-based surveillance; we implemented surveillance reactively in response to a clinical case of measles. Retrospective analysis of archival samples showed MeV RNA was not detected in wastewater samples before the first case was identified; possible reasons were RNA degradation in archived samples caused by handling and freeze–thaw cycles (*39*), sampling frequency, method sensitivity, or the rural nature of the sewershed where the outbreak occurred. 

In our study, WS for measles was robust, even though it was conducted in a community where a substantial portion of the population is served by septic systems. Septic systems and sewage hauling can influence the dynamics of measles signal in wastewater; weekly averaging or potential delays in the detection of MeV signals associated with septic hauling could obscure lead time. Our findings could likely be extrapolated to other viruses in different rural systems with similar sewage systems. Despite the possible influence of septic hauling on the temporal dynamics of wastewater signal, WS retained the ability to track disease at the community level, independent of traditional surveillance. We applied shedding rates calculated from a sewer lateral of a hospital with confirmed measles admissions to wastewater data to produce plausible estimates of measles cases. Investigation of MeV fractionation in wastewater indicates that most virus was found within the liquid phase of the wastewater matrix, consistent with MeV shedding primarily in urine and with previous work (*27*). Concentration of the supernatant may be most efficient for the purpose of WS for MeV. Our findings suggest that WS can act as an independent weekly measure of MeV at LPCC. We did not find that WS was a leading indicator of cases when data were aggregated by epidemiologic week. 

Despite its promise, WS for MeV has limitations. Early warning requires ongoing WS with sufficiently frequent sampling. Establishing and operating WS programs requires investment in infrastructure, personnel, and reagents. WS is less feasible in rural or remote communities lacking centralized wastewater infrastructure or relying on septic systems. WS for MeV is more effective in communities with undervaccinated populations and centralized WWTPs, where high transmission risk and actionable surveillance data align. Of importance, WS is not a replacement for clinical surveillance; it cannot identify infected persons, confirm diagnosis, or establish transmission chain. Assessing the accuracy of WS through comparison with reported clinical cases is flawed when clinical cases are likely underreported. Our study does not draw conclusions about the ability of WS to provide early warning of outbreak onset; retrospective analysis of archived samples is likely to miss early signals close to the assay limit of detection because of sample degradation. 

We indirectly estimated shedding rates on the basis of mean suspended solids values for hospital effluent and MeV partitioning in wastewater, so those estimates must be cautiously interpreted. Additional uncertainty in shedding rate estimates arises from the use of facility water for flow-rate estimation. The LPCC receives waste from residences served by septic systems. However, outside of the agrifood industry, most septic tanks are emptied infrequently. It is unlikely that households emptied septic tanks during the measles outbreak in a manner that contributed meaningfully to the MeV signal at the treatment facility. In addition, diapering of infected children reduces the MeV load at the WWTP and potentially influences the signal.

This investigation supports the continued use of WS for MeV; it complements routine case-based surveillance for measles. Despite the cost of establishing WS for MeV, it can provide a net positive investment for the healthcare system; the high cost of measles outbreaks means that preventing even a few cases through informed or early action could offset WS expenses (*21*). Once established, a WS system can be adapted to survey multiple pathogens for little additional cost with judicious selection of target pathogens and informed variation in sampling frequency. WS remains viable in circumstances in which clinical metrics may underrepresent actual cases and can inform public health services of changes in disease trajectory.

Appendix Additional information about wastewater surveillance used to track a resurgent outbreak of measles, Ontario, Canada, 2025.
